# Comparative Analysis of Surface Roughness and Plastic Deformation of Reciprocating Instruments after Clinical Use

**DOI:** 10.3390/ma17163978

**Published:** 2024-08-10

**Authors:** Ángel Herrera, Magdalena Azabal, Jesús R. Jimenez-Octavio, Juan C. del Real-Romero, Sara López de Armentia, Juan M. Asensio-Gil, Ana Arias

**Affiliations:** 1 Department of Conservative and Prosthetic Dentistry, School of Dentistry, Complutense University, 28040 Madrid, Spain; angelh03@ucm.es; 2 Department of Dentistry, School of Medicine, San Pablo CEU University, 28003 Madrid, Spain; m.azabal@gmail.com; 3 MOBIOS Lab, Institute for Research in Technology, ICAI-School of Engineering, Comillas Pontifical University, 28015 Madrid, Spain; joctavio@comillas.edu (J.R.J.-O.); delreal@iit.comillas.edu (J.C.d.R.-R.); sara.lopez@comillas.edu (S.L.d.A.); jasensio@comillas.edu (J.M.A.-G.)

**Keywords:** surface roughness, WaveOne gold, EdgeOne fire, profilometry, plastic deformation, nickel–titanium, root canal preparation, Ni-Ti file

## Abstract

This study assessed the surface topography and plastic deformation (PD) of new and used contemporary reciprocating instruments. Twenty-six WaveOne Gold (WOG) and EdgeOne Fire (EO) instruments were photographed under magnification. The instruments were randomly assigned to a control group of new instruments preserved for surface roughness analysis (n = 6 each), or to an experimental group to shape the root canal system of a single molar (n = 20 each), making a total of four groups (WOGnew, EOnew, WOGused, EOused). Used instruments were also photographed after instrumentation. The presence of fractures was registered. Preoperative and postoperative images were randomly ordered for evaluation. Two blinded calibrated examiners evaluated the presence of PD. Inter-observer agreement was calculated with the Kappa coefficient (K = 0.89). 3D profilometry was also used for the surface roughness analysis of six randomly selected instruments from the WOGused and EOused groups. Chi-square and two-way ANOVA tests were used to, respectively, compare PD and changes in surface roughness among the groups. No instruments fractured; however, a significantly greater percentage of EO instruments suffered plastic deformation than WOG instruments (*p* < 0.001), (OR = 11.09 (CI 95% 2.6–56.3)). The overall surface roughness was higher for most parameters in the EO instruments (*p* < 0.05). Single uses of EO instruments produced significantly higher chances of PD and increased surface roughness values compared to WOG.

## 1. Introduction

Nickel–titanium (NiTi) rotary instruments have been used as standard tools for root canal preparation since their introduction by Walia et al. in 1988 [1]. Better mechanical properties and increased flexibility make them a more suitable option when compared to stainless steel instruments, allowing clinicians to optimize endodontic procedures by reducing time and cost [2,3]. However, the possibility of unexpected fracture of these instruments due to cyclic fatigue (CF) or torsional failure (TF) is still a significant concern among clinicians [4]. For that reason, manufacturers have tried to improve the mechanical strength and performance of NiTi instruments for decades. Initial improvements consisted mainly of changes in the designs of the instruments, but over time, different motions and thermo-mechanical treatments of the raw NiTi alloy have also been incorporated [5]. One notable development was the introduction of the reciprocating concept [6]. In contrast to the traditional continuous clockwise rotation of these instruments, reciprocating motion includes a counterclockwise cutting movement and a clockwise release of the instrument, with a greater angle for the cutting than for the release function [7]. It was first proposed by Yared, who showed how a single NiTi instrument could shape an entire root canal more rapidly with a reciprocation motion [8]. This new concept of motion inspired manufacturers to develop specific instruments. The WaveOne system (WO) (Dentsply Maillefer, Ballaigues, Switzerland) was one of the first to be specifically designed for this purpose [9]. WO instruments are manufactured using M-Wire alloy, a pre-milling heat treatment process that alters the microstructure of NiTi alloy and has been demonstrated to produce more flexible and resistant instruments [10,11]. Along with the introduction of more flexible and resistant reciprocating instruments, the “single-use single-file” shaping concept was also incorporated, suggesting the use of a single instrument to shape the root canal from start to finish [12]. This concept simplified endodontic procedures by reducing the number of instruments needed for canal preparation. The WO system evolved with the introduction of WaveOne Gold (WOG) (Dentsply Sirona, Ballaigues, Switzerland) in 2015, which uses the same kinematics but incorporates a special post-milling heat treatment, becoming known as the ‘Gold alloy’ as a result of the gold-like surface layer produced due to the heating and cooling processes used during its production. This additional thermal treatment modifies the crystal microstructure of the alloy, transitioning from an austenitic (cubic) phase, which is more rigid, to a martensitic (tetragonal) phase, characterized by its flexibility and ductility [5], providing the instrument with greater flexibility and increased resistance to CF [13].

However, despite the numerous efforts over the years to improve the mechanical properties of endodontic instruments, a recent narrative review highlighted future directions in the manufacturing of low-cost systems as an alternative for root canal shaping [14]. It has been demonstrated that the reliability of low-cost instruments might be compromised [15], and mechanical and morphological properties may be negatively affected if the instrument is reused, which is a common practice among clinicians [15,16]. The EdgeOne Fire (EO) (EdgeEndo, Albuquerque, NM, USA) system mimics the concept and morphological characteristics of WOG instruments. It has the same cross-section and taper, uses the same sequence, and is meant to be used in the same reciprocating movement with the same parameters as WOG, but is available at a lower price [12]. The main difference between both systems is the heat treatment, which is conducted with a proprietary alloy called Fire-Wire that is supposedly more resistant to CF [17]. In fact, it has been reported that the largest segments of the specialist endodontic instrument market belong specifically to these two manufacturers, with 56.9% belonging to Dentsply Sirona and 28.8% to EdgeEndo [15].

At the same time, even though instruments manufactured with martensitic alloys have demonstrated better mechanical properties [5], they may deform more easily than those manufactured with conventional NiTi alloys due to their lower elastic modulus, and consequently, shaping effectiveness would decrease after a limited number of uses with the deterioration of the instrument. In fact, it has been reported that severe plastic deformation and aging contribute to raising the martensite transition temperature in shape memory alloys submitted to post-processing heat treatment procedures [18].

A widely accepted method for determining the deterioration of reciprocating instruments is the measurement of roughness parameters [19,20,21]. In fact, surface topography parameters are considered crucial indicators of instrument safety during clinical use. Surface roughness determines the texture of the surface of the instrument, identifying small-scale irregularities and deviations from smooth surfaces. Since instrument fractures typically start in cracks originating from defects, instruments with low surface roughness exhibit high CF resistance [22,23]. Firstly, if the finishing of an instrument is non-optimal and surface roughness is high, detritus can accumulate during use and reduce the cutting efficiency of the instrument [24]. Secondly, surface irregularities could act as the starting point of cracks that could lead to deformation and fracture after accumulated fatigue [22]. These irregularities can result from the manufacturing process or from clinical use, where wear and tear create additional roughness. Moreover, changes in surface curvature are closely associated with plastic deformation (PD) [25]. PD is a type of permanent deformation produced when the material is subjected to stress beyond the elastic limit, for example, due to the forces experienced during root canal preparation [20]. Repeated clinical use may produce PD.

For all these reasons, it is important to understand how morphological parameters, such as PD and surface roughness, change in different types of instruments (including low-cost instruments) after use, so that users have the knowledge to discard an instrument if a safe and effective performance cannot be guaranteed when it is reused. Therefore, the aims of this study were to assess the surface topography and PD of contemporary reciprocating instruments (WOG and EO) and to evaluate the influence of clinical use on these variables.

## 2. Materials and Methods

### 2.1. Sample Size Calculation

The sample size was calculated based on data regarding the surface roughness (Ra) of unused and used reciprocating instruments from a previously published study by Moreira et al. [26]. Accepting an alpha risk of 0.05 and a power of 0.8 in a two-tailed test, 5 specimens per group are necessary to recognize a difference greater than or equal to 0.18 units as statistically significant if a common standard deviation is assumed to be 0.1.

### 2.2. NiTi Instruments Used

In total, 26 WOG Primary and 26 EO Primary instruments (apical size 25.07v taper) were used for the study. All instruments were photographed at ×10 and ×30 magnification using a digital camera, the Leica EC3 (Leica Camera, Wetzlar, Germany), mounted on a stereoscopic microscope, the Nikon SMZ800 (Nikon Corp., Minato, Japan). If any defect was detected, the instrument was discarded. Each instrument was randomly assigned to a control group of new instruments (n = 6 each) or to an experimental group to shape the root canal system of a single molar after glide path preparation (n = 20 each), making a total of 4 groups: WOGnew, EOnew, WOGused, and EOused. The instruments assigned to the WOGnew and EOnew groups were preserved for a topography analysis.

### 2.3. Clinical Procedure

Instruments assigned to the WOGused and EOused groups were randomly ordered to be used for root canal treatments performed on first mandibular molars. A specialist with more than 30 years of experience performed the treatments. Mandibular molars presenting previous endodontic treatment, incomplete root formation, highly calcified canals, or severe radiographic curvatures were excluded. Root canal treatments were performed under magnification with an operating microscope (Zumax Medical, Suzhou, China). After establishing coronal access, patency was obtained with a 10-K file (Dentsply Maillefer, Ballaigues, Switzerland). The working length was determined with an apex locator Justy II (Yoshida Dentcraft, Tokyo, Japan) and confirmed radiographically. The WaveOne Gold Glider (Dentsply Sirona, Ballaigues, Switzerland) instrument was used to obtain a glide path. Then, the canals were shaped with the corresponding reciprocating file and the endodontic motor X-Smart iQ (Dentsply Sirona, Ballaigues, Switzerland) using the reciprocating program WAVEONE ALL, following the manufacturer’s directions for use. Five percent NaOCl was used for irrigation during the entire shaping procedure. Once the instrumentation was completed, all files were cleaned with a sterile gauze soaked in alcohol, packaged individually, and sterilized in an autoclave (Quirumed, Bunzl, London, UK) set to a temperature of 134 °C and 210 KPa of pressure for 30 min.

### 2.4. Analysis of the Instruments

#### 2.4.1. Analysis via Stereoscopic Microscopy

The used instruments were then photographed again at the same magnification. Preoperative and postoperative images of the instruments were randomly ordered for evaluation via a Keynote (Apple, Cupertino, CA, USA) presentation, and color was removed to prevent potential visual recognition of the different alloys. The presence of PD was evaluated during the presentation. Two independent, blinded examiners independently registered in an Excel document if a deformation was observed in any of the images. The examiners had been previously calibrated to ensure consistency and reliability in their assessments. This calibration was conducted using a smaller dataset of selected images from the larger study dataset. Inter-observer agreement was calculated using the Kappa coefficient. In the event of a disagreement, both examiners discussed until they reached a consensus. In the images where a deformation was observed, the distance from the tip of the instrument to the defect was measured by another operator. The presence of fractures in the instruments was also registered.

#### 2.4.2. Analysis via Non-Contact 3D Profilometry

The 6 new instruments in groups WOGnew and EOnew and 6 used instruments randomly selected from those with no PD in the WOGused and EOused groups were analyzed with a 3D Optodigital Microscope DSX 1000 (Olympus, Tokyo, Japan) to detect changes in surface roughness at a set of points. The four cutting blades and adjacent flutes were analyzed at varying distances from the tip of each instrument (3 mm, 5 mm, and 12 mm) with a ×10 lens and ×140 zoom and a ×40 lens and ×1600 zoom, respectively, for the blades and the flutes. The standard ISO 21920-2:2021 [27] and 25178-2:2021 [28] were followed to determine the roughness of the flutes and blades of the instruments. These standards provide the framework for measuring the surface texture of an item using 2D and 3D profiling techniques, respectively. The Arithmetic Mean Roughness (Ra), Root Mean Square Roughness (Rq), and Average Maximum Height of the Profile (Rz) of the cutting blades were calculated. Ra was calculated by determining the mean of the absolute values of surface deviations from a mean line over a predefined measurement length. Rq was determined by calculating the root mean square of the surface deviations from the mean line over the sample length. Rz measures the average peak-to-valley height within a series of sampling lengths. Analogous 3D versions of the Arithmetic Mean Surface Roughness (Sa), Root Mean Square Surface Roughness (Sq), and Maximum Height of the Surface (Sz) parameters were calculated in an area instead of on a line for the flutes of the instruments. Metrics were compared between new and used instruments, and the ratio of new/used instruments was also calculated to assess the impact of clinical use on surface roughness.

#### 2.4.3. Data Analysis

IBM SPSS Statistics 29.0 (SPSS Inc., Chicago, IL, USA) software was used for statistical analysis. The chi-square test was used to compare plastic deformation between the two systems, and the odds ratio (OR) was calculated. A two-factor ANOVA was used to compare surface roughness between both systems in both groups after verification of the normal distribution of data.

## 3. Results

### 3.1. Plastic Deformation

**Figure 1:**
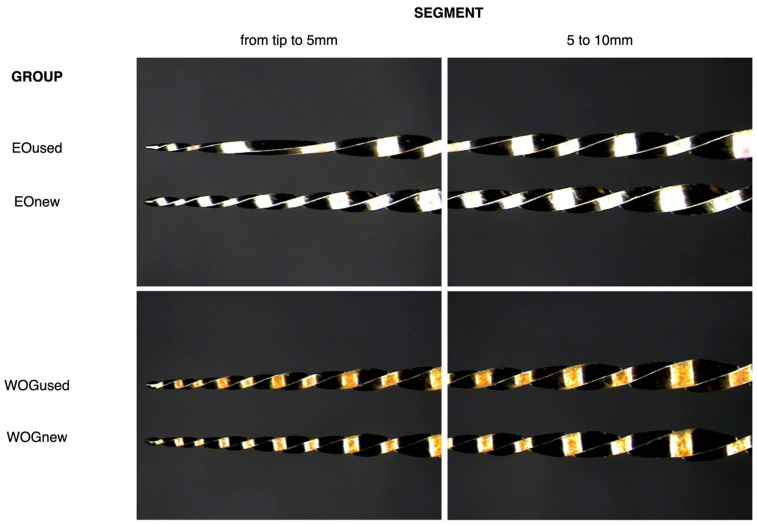
Stereoscopic microscopy photographs of new and used EO and WOG at two different segments of the instruments. Notice the plastic deformation in the apical portion of the EOused instruments.

Inter-observer agreement was very high (K = 0.89). No instrument was fractured during the shaping procedure. A significantly higher percentage (*p* < 0.001) of EO instruments suffered plastic deformation (60%) in comparison with WOG (10%) (Figure 1), with an odds ratio (OR) of 11.09 (CI 95% 2.6–56.3). Additionally, the distance from the tip to the plastic deformation start site varied between both reciprocating systems (mean (SD) = 2.03 (0.9) mm and 4.04 (1.6) mm from the tip, respectively, for EO and WOG). Figure 1 shows the effects of clinical use on both types of instruments.

### 3.2. Surface Roughness

Table 1 shows the mean (standard deviation (SD)) surface roughness parameters for new and used EO and WOG instruments in both the flute (Sa, Sq, and Sz) and the blade (Ra, Rq, and Rz) at the three apical thirds (apical, middle, and coronal). Ratios between used and new instruments are also included for each parameter.

**Figure 2:**
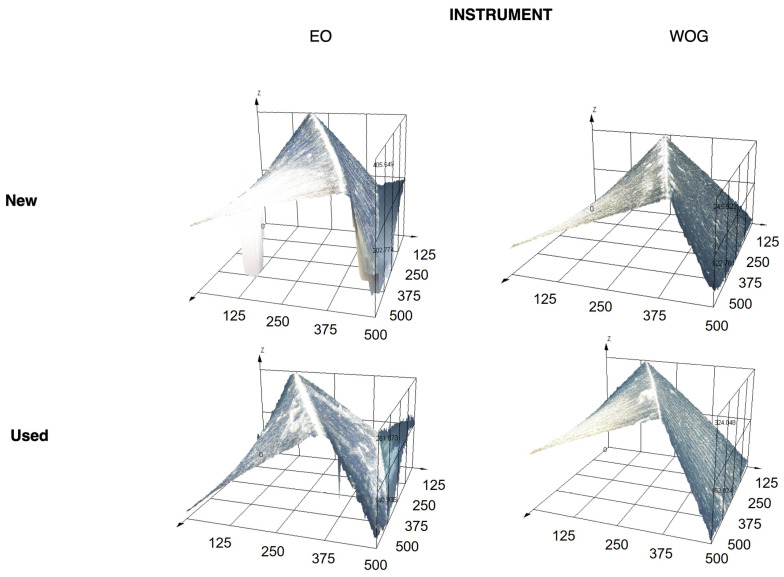
Representative three-dimensional surface plot measurements (in microns) of new and used EO and WOG instruments.

Significant statistical differences were observed between the WOG and EO instruments in terms of the Ra (*p* = 0.007) and Rq (*p* = 0.015) parameters in the apical portion, where the EO instruments suffered a greater increment in roughness after clinical use compared to WOG. No statistical differences were observed in Ra and Rq at the middle and coronal levels, or in the Rz, Sa, Sq, and Sz parameters (*p* > 0.05). At the same time, the ratio between new and used instruments was higher for EO instruments for most measurements at all levels, indicating more variability compared to WOG, except for Sz, where WOG had greater values. Specifically, the EO instruments had higher ratios in both the apical and coronal portions for all measurements except Sq and Sz, while the WOG instruments only showed higher ratios in the middle portion, except for Sa and Sq. Figure 2 shows representative 3D surface plots of new and used instruments. New WOG and EO instruments show smooth, polished surfaces with fine, parallel machining marks and consistent light reflection. In contrast, the used files exhibit significant surface roughness, with prominent scratches and wear marks, notable peaks and valleys close to the cutting edge, and inconsistent light reflection, highlighting that the impact of wear and deformation from use is more highly accrued in EO than in WOG instruments.

**Figure 3:**
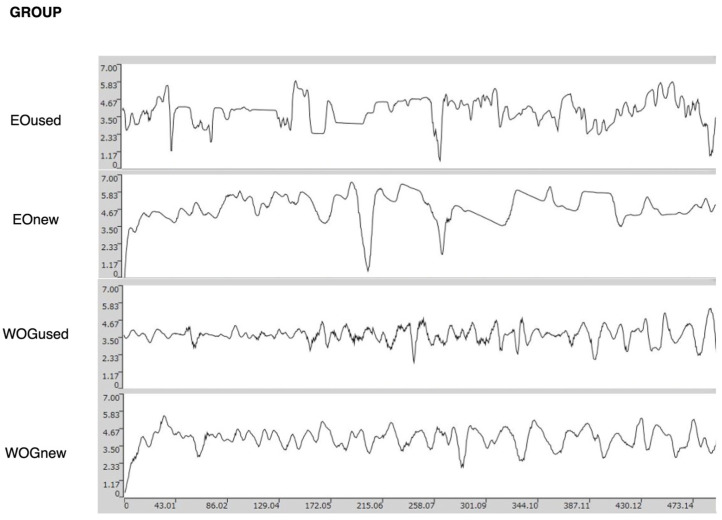
Roughness profiles (in microns) of the blades of representative samples of new and used EO and WOG instruments.

To elucidate the homogeneity of the surface, Figure 3 shows the roughness profiles of representative samples of new and used WOG and EO instruments. The more heterogeneous profiles of both new and used EO instruments can be noticed due to their higher peaks and valleys.

## 4. Discussion

The presence of surface irregularities on NiTi rotary instruments, whether from their manufacturing process or clinical use, may serve as starting points for cracks, which can ultimately lead to instrument breakage [29]. Moreover, they can also affect the efficacy of the instruments [22]. Several methods are currently available to evaluate these properties quantitatively and qualitatively. Stereoscopic microscopy, introduced more than 30 years ago [30], has been used previously to assess the integrity of endodontic instruments before and after clinical use. It is a valuable method for understanding the macro-level features of endodontic instruments [31,32], with reasonable cost effectiveness, quick sample preparation, and ease of use. All these reasons make it a valid tool of choice for the analysis of PD if the evaluator has been adequately calibrated. In the current study, two blinded examiners independently evaluated the existence of PD in randomly ordered images of new and used instruments after calibration, with a high level of agreement.

Nevertheless, more complex measuring systems are required in order to understand surface topography in detail. While scanning electron microscopy (SEM) is a commonly employed technique for determining alterations in instrument surfaces after use [33,34], the bidimensional images obtained are inadequate for complete topography analyses and quantitative comparisons of endodontic instruments [35]. Alternatively, atomic force microscopy (AFM) has been proposed for scenarios requiring three-dimensional measurements. Various studies have utilized AFM to evaluate the surface roughness of endodontic files [29,30]. However, this technique can only measure small surfaces (around 20 µm × 20 µm), significantly reducing the sample representativity [36]. Additionally, this method requires an ultra-flat surface, which is not the case for endodontic instruments. For all these reasons and to complement the results from the PD analysis, the present study opted for a non-contact three-dimensional optical profilometry analysis to evaluate surface roughness. This non-invasive technique reduces the risk of modifying surface properties, thus saving significant amounts of time and resources and permitting the obtention of quantitative data while scanning wider 3D areas than AFM. Multiple measurements can also be obtained from the same sample even if irregular surfaces are present, making it optimal for evaluating endodontic instruments [21,35].

Although it had been previously used in other dental fields [36,37], the use of optical profilometry in endodontics was first proposed by Ferreira et al. [21], who analyzed an endodontic instrument using the Sa, Sq, and Sz parameters. When evaluating the surface roughness of endodontic files, the utilization of international standards is paramount. The current study followed the latest ISO guidelines, which suggest additional parameters for surface roughness evaluation (Ra, Rq, Rz, Sa, Sq, and Sz), offering a more comprehensive evaluation of surface textures and of the deterioration that instruments suffer with clinical use [19,20,21].

The impact of roughness parameters may affect the clinical efficacy of endodontic files from different perspectives. Ra provides a fundamental understanding of surface texture in the cutting blade. Rz represents the surface smoothness and helps detect extreme variations in surface topology. Analogous 3D versions of these parameters explain the behavior of the flutes [27,28]. The results of the present study show how new instruments present smoother surfaces than used instruments. The wear of the instruments during use may affect their performance. Both types of endodontic files experienced some degradation with use, characterized by increased roughness and surface irregularities; however, the specific patterns and amounts of scratches and material deformation differ, perhaps due to the material compositions or manufacturing processes.

The current study showed how the EO instruments suffered a greater increment in roughness after clinical use compared to WOG in the apical portion. At the same time, no significant differences were observed in the middle and coronal levels, suggesting that the apical region is more susceptible to wear and degradation. The excessive roughness produced by the deterioration of instruments with use can lead to debris accumulation in the instrument, a reduction in cutting efficiency during root canal preparation, and a higher probability of instrument fracture and, hence, might directly impact the clinical outcome if the instrument is reused [38]. With these findings in mind, it seems that WOG would be more predictable than EO instruments in the event of reprocessing for a second clinical use.

Understanding and optimizing these parameters is, thus, essential for engineering endodontic files that are not only efficient and effective in their application, but also durable and safe for clinical use. By adhering to these standards, this study contributes to a better understanding of how surface roughness affects the performance of endodontic instruments.

Although one of the main drawbacks of NiTi rotary instruments is the potential for unexpected fracture inside the canal [39], and the CF resistance of these instruments is of interest, the literature has already provided valid data on the CF behavior of both instruments analyzed in the present study [18,38,39,40,41,42,43]. In contrast, surface roughness and PD data remained to be determined. For this reason, the current study aimed to analyze morphological parameters that are more relevant for martensitic instruments, such as PD and surface roughness. A notable strength of this study is that the two systems used have an almost identical design, minimizing morphological variability and providing more accurate analyses. No PD was observed in the new set of instruments. However, there was a significant difference between the files after clinical use. Despite both systems being relatively similar, certain dissimilarities may account for the observed variation in deformation behavior [44,45]. According to Martins et al. [32], EO displays more surface irregularities under high magnification than WOG, which, in addition to its lower core diameter, might make the instrument more prone to deformation under stress. In fact, this was found to be the case for EO, as it demonstrated a tendency to deform easily even after a single use in the current study. While CF resistance is spotlighted in the evaluation of endodontic instruments, reliability extends beyond CF to include each instrument’s consistency across its intended lifespan, preserving its shape and cutting efficiency [39,46]. Higher PD could mean a warning for potentially compromised performance in clinical scenarios.

It is worth mentioning that the behavior of instruments is also dependent on temperature. The utilization of differential scanning calorimetry (DSC) in evaluating NiTi instruments plays an essential role in comprehensively understanding their mechanical properties at different temperature conditions and ranges [47,48]. It has been reported that WOG instruments exhibit a martensitic phase at room temperature and transform into austenite at temperatures higher than body temperature, while FireWire instruments exhibit variable phase transformations with temperature changes, indicating a potential for more variable behavior under different clinical conditions [48]. However, even if made from alloys with similar compositions, previous authors have suggested that the differences in physical properties could be attributed to the processing and manufacturing of the instruments rather than their metallurgical composition alone [49,50].

As previously mentioned, concerns regarding the potential manufacture of low-cost systems as an alternative for root canal shaping have been raised [14]. Variable quality could be encountered between instruments [32,48], and mechanical and morphological properties may be negatively affected, especially if reused. This situation worsens because both systems have been designed explicitly following the single-file philosophy, where a single instrument shapes the canal from the beginning to the end without using additional files. Therefore, this concept potentially leads to more instrument stress and deformation. This is particularly true for martensitic files and is consistent with the results obtained in this study.

Few previous studies have analyzed the effects of instrumentation on WOG. However, the methodological heterogeneity among studies implies significant challenges in comparing research findings, since some studies traditionally focus on a limited set of parameters (such as Ra). According to Zafar et al., WOG displayed the highest surface roughness before and after instrumentation among the files tested [38]. Previous studies also found that WOG showed higher surface porosity than WO [51], but none have reported on the effects of the clinical use of low-cost instruments, especially with such a broad range of parameters (Ra, Rq, Rz, Sa, Sq, Sz). Nevertheless, previous studies found that clinical use affected the surface roughness of Reciproc R25, reducing it after use [26]. Although apparently counterintuitive, this could be explained by the possible wear produced during the shaping procedure. This would lead to the eroding of the surface irregularities, polishing the surface.

The present study provides additional insights into deviations in the surface textures of different types of instruments after clinical use. Although a potential limitation of the study is that highly complex root canal anatomies, such as double curvatures or highly calcified canals, were not included, clinicians tend to discard instruments after shaping such difficult anatomies. For this reason, information on the effects of clinical use is of less clinical value in these circumstances. A more relevant limitation could be the single use evaluation, since the wear and degradation of endodontic instruments may not be linear, and the single use data might not represent the cumulative effect of multiple uses, which, as previously mentioned, is a common practice in clinical settings [15]. Thus, although it may be challenging to correlate these findings with real-life scenarios, this study provides valuable information about the effects of clinical use on WOG and EO instruments. While a multiscale analysis with the inclusion of some other parameters might offer additional insights into the surface characteristics of the instruments before and after use, the findings of the present study indicate that clinical use affects the performance of EO and WOG instruments differently. EO instruments exhibited more significant changes in roughness after clinical use compared to WOG instruments. Coupled with the higher rates of PD observed in the EO instruments, and with the heterogenous surface profile (Figure 3), this study suggests that WOG would perform more uniformly and predictably if the instruments were reused. Comparative studies of instruments that consider factors such as lifespan, performance, and, ultimately, long-term clinical outcomes are necessary to establish more comprehensive performance benchmarks and guide clinicians in selecting the most efficient and reliable instruments. In summary, WOG offers greater stability and predictability in its mechanical properties than EO, mainly due to its consistent material behavior and decreased deterioration after use.

## 5. Conclusions

Within the limitations of this study, it can be concluded that clinical use affected the EO and WOG instruments differently. The EO instruments exhibited significant plastic deformation and increased roughness in the apical portion when compared to WOG after the root canal preparation of a single molar.

## Figures and Tables

**Table 1:** Mean (μm) and standard deviation (SD) surface roughness parameters for new and used EO and WOG instruments in both the flute (Sa, Sq, and Sz) and the blade (Ra, Rq, and Rz) at the three apical thirds (apical, middle, and coronal), as well as ratios between new and used instruments for each parameter.

		Flute									Blade								
		Average Roughness (Sa)			Quadratic Avarage (Sq)			Height (Sz)			Average Roughness (Ra)			Quadratic Avarage (Rq)			Height (Rz)		
Instrument	Portion	New	Used	Ratio	New	Used	Ratio	New	Used	Ratio	New	Used	Ratio	New	Used	Ratio	New	Used	Ratio
WOG	Apical (3 mm)	1.2 (0.48)	1.4 (0.08)	1.17	1.53 (0.52)	1.82 (0.81)	1.18	9.4 (2.86)	12.05 (1.5)	1.28	3.29 (0,27)	3.14 (0.29)	0.95	3.99 (0.28)	3.91 (0.3)	0.98	16.56 (0.72)	17.86 (0.91)	1.08
EO		0.84 (0.19)	1.18 (0.61)	1.40	1.11 (0.21)	1.58 (0.04)	1.42	8.27 (0.2)	10.56 (1.32)	1.28	2.68 (0.12)	3.63 (0.3)	1.35	3.28 (0.19)	4.32 (0.41)	1.32	13.23 (0.51)	15.98 (1.2)	1.21
WOG	Middle (5 mm)	1.25 (0.57)	1.26 (0.1)	1.01	1.57 (0.63)	1.63 (0.22)	1.03	9.21 (2.5)	10.86 (3.19)	1.18	2.78 (0.34)	3.86 (0.84)	1.39	3.45 (0.4)	4.67 (0.88)	1.35	15.5 (0.98)	19.96 (3.01)	1.29
EO		0.97 (0.1)	1.06 (0.15)	1.09	1.27 (0.14)	1.41 (0.16)	1.11	8.69 (1.83)	9.94 (1.16)	1.14	2.12 (0.3)	2.71 (0.5)	1.28	2.55 (0.38)	3.2 (0.49)	1.25	10.44 (1.76)	12.94 (1.51)	1.24
WOG	Coronal (12 mm)	0.84 (0.12)	1.07 (0.12)	1.28	1.07 (0.1)	1.44 (0.21)	1.34	7.49 (0.72)	11.38 (3.65)	1.52	3.9 (1.67)	3.36 (0.38)	1.39	4.9 (2.24)	4.24 (0.41)	0.86	23.37 (11.4)	18.92 (1.32)	0.80
EO		0.9 (0.11)	1.16 (0.1)	1.29	1.12 (0.15)	1.43 (0.11)	1.28	7.46 (0.57)	9.97 (0.97)	1.34	1.9 (0.73)	2.24 (0.53)	1.17	2.45 (1.05)	2.68 (0.6)	1.09	11.16 (4.82)	11.06 (2.56)	0.99

## Data Availability

The raw data supporting the conclusions of this article will be made available by the authors on request.
